# Reduction of COVID-19 Anxiety Levels Through Relaxation Techniques: A Study Carried Out in Northern Spain on a Sample of Young University Students

**DOI:** 10.3389/fpsyg.2020.02038

**Published:** 2020-08-25

**Authors:** Naiara Ozamiz-Etxebarria, María Dosil Santamaría, Amaia Eiguren Munitis, Maitane Picaza Gorrotxategi

**Affiliations:** ^1^Department of Developmental and Educational Psychology, University of the Basque Country UPV/EHU, Leioa, Spain; ^2^Department of Research and Diagnostic Methods in Education, University of the Basque Country UPV/EHU, Leioa, Spain; ^3^Department of Didactics and School Organization, University of the Basque Country UPV/EHU, Leioa, Spain

**Keywords:** anxiety, relaxation techniques, university students, telematic psychoeducation, COVID-19

## Abstract

Since March 14, 2020, Spain has been in a state of alarm due to the crisis created by the outbreak of COVID-19. This measure has led to strict levels of lockdown. This situation has led to an increase in anxiety levels among the younger population. For this reason, an intervention was carried out on university voluntary participants in order to help lower their anxiety levels. Specifically, a telematic workshop was implemented to teach emotional literacy and relaxation techniques combined with the practice of the techniques in an autonomous manner. Anxiety measurements were taken before and after the workshop using the Generalized Anxiety Disorder – 7 (GAD-7) scale. The results show that Jacobson’s progressive relaxation techniques, Schultz’s autogenic training, abdominal relaxations, and visualizations are effective in lowering the anxiety levels of university students as an alternative to pharmacotherapy.

## Introduction

The Spanish State has been in a state of alert since March 14, 2020 due to the COVID-19 pandemic. As demonstrated in a recent investigation, the general population is experiencing situations of anxiety, and in some groups and individuals, the level of anxiety has increased due to lockdown and uncertainty and due to the de-escalation situation we are experiencing (Hao et al., 2020).

Contrary to initial estimates, this historical and unprecedented situation for the younger population is affecting them psychologically more than the adult population (Taylor et al., 2008). In particular, students are proving to be one of the most vulnerable populations due to the age of near adolescence when peers become more important (Gallego et al., 2011). The World Health Organization (World Health Organization (WHO), 2018) understands that adolescence occurs between the ages of 10 and 19 years in its two phases, early adolescence (10–14 years) and late adolescence (15–19 years). The situation is aggravated by the stress to which they are subjected by the studies themselves linked to the new methodologies of receiving classes telematically and information and communication technologies (ICTs) (Jung et al., 2012; Rogero-García, 2020).

For all these reasons, the students refer to living insomnia in the face of changes in routine and stress that this new situation may be causing them, in addition to experiencing high levels of anxiety characterized by feelings of catastrophe or imminent danger, feelings of risk, tension, insecurity, and suffering.

Against this background, several studies are showing that telematic psychotherapy can be of great help to people who are suffering psychologically with this pandemic (Macias and Valero, 2018). In the Basque Country, psychological services were set up in the Basque Health Service (Osakidetza) from the moment that COVID-19 cases began to increase. However, not all the population has access to this resource; thus, it is important to provide psychological resources by other means, such as guidelines for dealing with anxiety in the face of the pandemic.

According to the Anxiety Disorders Association of America and the Spanish Society for the Study of Anxiety and Stress, there are different methods for dealing with anxiety. Non-pharmacological interventions, such as relaxation, yoga, problem-solving techniques, biofeedback, psychotherapy, and cognitive–behavioral techniques, are the most effective in combating anxiety (Bushell, 1998; Vaccaro et al., 2019; Armat et al., 2020; Gadea et al., 2020).

There are many physical and psychological benefits that can be achieved through the practice of relaxation techniques (Smith et al., 2018):

• They help to create feelings of well-being and tranquility as well as help to care for sleep hygiene and combat insomnia.
• They strengthen the immune system, help to lower anxiety, and increase the ability to cope with different situations that can create stress.
• They decrease physiological symptoms that can occur with anxiety, such as decreased heartbeat and breathing and increased ability to concentrate and memory.
• They increase energy level, positive thoughts, and creativity and help to create a positive self-image.

In summary, through relaxation, positive effects can be achieved on three different levels. Firstly, on a physiological level, muscle tension and breathing frequency are reduced, and heart rate is lowered. Secondly, on a behavioral level, controlled and calm movements and hypoactivity are achieved. Finally, on a cognitive level, there are thoughts of tranquility, well-being, images of calm situations, and perception of sweet feelings (Rasid and Parish, 1998).

According to Burgos Varo et al. (2006), the most effective relaxation techniques to reduce the levels of autonomic activation and the unpleasant experience of anxiety by improving sleep and other stress-related symptoms are Jacobson’s progressive relaxation (Bushell, 1998), Schultz autogenic training (Schultz, 1969), breathing techniques, and visualizations as explained below.

Jacobson’s progressive muscle relaxation is a technique in which the muscles are first tensed and then relaxed by different muscle groups throughout the body. The main objective is to recognize the difference between a state of muscle tension and a state of muscle relaxation. In this way, a state of muscular relaxation is achieved that progressively becomes generalized to the whole body (Mármol, 2013; Toro, 2019).

In Schultz’s autogenic training, there are six exercises that must be learned progressively. These exercises consist of the passive concentration of the body’s own sensations. Through some simple self-instructions, the person will be able to get his extremities first, and the rest of his body later, to relax through the sensations of heat and weight. The regulation of the heart or breathing frequency must be the result of a process that must occur automatically, after thinking that the heart beats calmly or that breathing is relaxed. Finally, the imagination of coolness in the head should help to have feelings of well-being and also clarity (Schultz, 1969).

Abdominal or diaphragmatic breathing is a breathing technique that consists of taking advantage of the empty space left by the abdomen as it expands forward to widen the lungs, since the diaphragm tends to go downward in such expansion (Chen et al., 2017; Rygiel, 2019). This method is based on the influence of the physiological component of emotions. It lowers blood pressure and heart rate and results in increased oxygenation of the cells, improved metabolism, and improved circulation through abdominal breathing. In addition to burning body fat, it strengthens lung health. Tiredness and poor ventilation can lead to energy deficits and depression; thus, practicing abdominal breathing also improves mood. It is a relaxation that provides multiple benefits: it provides more energy, improves digestion and skin appearance, helps control emotions and reduce fatigue, and improves anxiety symptoms.

Finally, visualization is a technique by which stress and anxiety can be reduced to bring the body and mind into a deep state of relaxation. Through this technique, a series of imaginary images is related to positive emotions. In this way, negative thoughts are transformed to reach a state of optimal well-being (López et al., 2019).

The aim of the present study is to evaluate the levels of anxiety in university students in the face of the COVID-19 pandemic crisis before and after telematic teaching and practice of different relaxation techniques. Their anxiety levels are expected to drop considerably.

## Materials and Methods

### Participants

In the first sample, 48 young people participated; four were eliminated from the total sample because they did not carry out the relaxations and, therefore, did not comply with the methodological criteria of this research.

Among a total of 44 participants, 7 (*n* = 15.9%) were men, and 37 (*n* = 84.1%) were women. Their average age was 19.5 years with an age range of 19–21 years. All of them were second year university students of the Degree in Child Education of the Faculty of Education of the Public University of the Basque Country (Leioa, Spain) to work as teachers in the age range of 0–6 years old. The criteria for inclusion were to take the subject Developmental and Learning Difficulties in the grade of Child Education, to commit to participate in the learning of relaxation techniques voluntarily, and to have a computer to be able to receive the training telematically. Regarding the criteria for exclusion, it was recommended that people who had any illness that prevented them from performing relaxation should not participate in the study.

The students participated voluntarily, received information about the research procedure, and gave their consent before participating in the study. Therefore, the procedure followed is approved by the Ethics Committee in accordance with the Declaration of Helsinki of the World Medical Association.

### Measures and Instruments

A brief introduction of socio-personal data and data of interest to this study was made before starting with the validated scale. Among the socio-personal data, age and sex were collected.

The scale administered was the scale for generalized anxiety disorder. The original version is the Generalized Anxiety Disorder – 7 (GAD-7) scale (Spitzer et al., 2006). It is an instrument created to screen for generalized anxiety disorder. This instrument is composed of seven Likert-type response items from 0 to 3 that include the symptoms and disability associated with the disorder. For its correction, a total score is obtained from the sum of scores of all items, which can range from 0 to 21. For the Spanish version that was validated with 212 people (García-Campayo et al., 2010), a Cronbach’s alpha coefficient of 0.93 was obtained. In the present research, Cronbach’s alpha was 0.80 in the pre-test and 0.74 in the post-test.

### Procedure

The first step was to get permission from the university’s Ethics Committee (UPV) to carry out this research.

The project was carried out at the University of the Basque Country (UPV/EHU) located in the north of Spain and with a world ranking of the 500 best universities. The students of the Infant Education degree participated in the project, specifically the students of the subject of Developmental and Learning Difficulties, which is taught in the second quarter of the second year (2020/2021).

This is a design without a comparison group, since the effectiveness of the relaxation techniques has been compared before performing and explaining the relaxation techniques and after the intervention.

### Workshop Procedure

In the first week of April, students were informed about the study. Those who decided to participate voluntarily completed the pre-tests using the Google Forms platform. The questionnaires completed by the students were anonymous and had to include a code to identify the relationship between the questionnaires completed before and after the workshop.

The training program consisted of four sessions led by a health psychologist with extensive experience in relaxation techniques (Lether, 1996**;** Lsalt and Kerr, 1997**;** Solomen and Aaron, 2015**;** Abuín, 2016). The first session focused on awareness of emotions and anxiety symptoms. The second session focused on learning relaxation techniques (Jacobson’s progressive relaxation, Schultz’s autogenic training, abdominal breathing, and visualization techniques). They were also advised to practice the different techniques and then choose the most appropriate one to lower their anxiety. The third session focused on learning the importance of practicing these techniques, and they shared experiences choosing the most appropriate technique for each one. After that, they practiced the chosen technique individually for 20 min for 2 weeks in their homes. The fourth session focused on the assessment of the techniques practiced by means of a group discussion (Table 1). All the sessions were carried out using the Blackboard Collaborate telematics program. The psychologist made an individualized follow-up of all the participants to make sure that they attended all the training sessions and performed the relaxation techniques. Once the workshop was completed, the anxiety measurements were taken again.

**TABLE 1:** Organization of the sessions and their systematization.

Telematic session	Objectives	Contents
1. Emotional education	To study in depth the concept of emotion and anxiety	Define emotions and their physiological, cognitive, and behavioral components Define anxiety and its disorders
2. Learning about relaxation techniques	To learn different relaxation techniques and their benefits	Jacobson’s progressive relaxation Schultz’s autogenic training Abdominal breathing Visualization techniques
3. Sharing first experiences of relaxation practice and understanding the need to practice such relaxation	To identify the most effective technique for each student and reading the article	Each student shared experiences of the techniques that helped them most An article was read that referred to the importance of learning these techniques (Justo, 2008)
4. Sharing of different experiences and appreciation of relaxation techniques	To share and assess techniques	A discussion group was held to share experiences about the practices they had carried out

### Data Analysis

The data extracted from the Google Forms questionnaire in Excel format were imported into the SPSS v.25 statistical program for further analysis. With the data obtained, descriptive analyses of the sample were carried out both on the socio-demographic data and on the differences between the pre- and post-relaxation technique tests. We categorized them into two groups, taking into account the mean of the total scale plus one standard deviation, so that all participants who were below that score were considered as not symptomatic (categorized with 1) and those who showed higher scores were considered as symptomatic (categorized with 2). The significant differences for these related samples were then analyzed using the non-parametric tests (taking into account the Shapiro–Wilk normality tests).

## Results

Among the relaxation techniques, 43.2% (*n* = 19) performed Jacobson’s relaxation techniques, 36.4% (*n* = 16) abdominal breathing techniques, 11.4% (*n* = 5) Schultz’s autogenic training, and 9.1% (*n* = 4) visualization. There was no significant difference in reducing anxiety levels according to the relaxation techniques that had been chosen.

As can be seen in Table 2, the mean anxiety measured with the GAD-7 scale is higher before relaxation techniques. In addition, a mean of more than 1 is observed at both times.

**TABLE 2:** Descriptive data in the pre- and post-anxiety tests (GAD-7) categorized.

	Pre	Post
N	44	44
M	1.5	1.3
DT	0.50	0.45
Minimum	1.00	1.00
Maximum	2.00	2.00

*Scores above one indicate anxiety.*

### Pre- and Post-test Anxiety Frequencies

Overall, 45.5% (*n* = 20) of the university students indicated that they felt anxiety during these moments of confinement due to the COVID-19, and 54.5% (*n* = 24) did not feel anxiety. Likewise, once the relaxation techniques were performed and again evaluated with the same scale, anxiety seemed to decrease considerably, with 72.7% (*n* = 32) not feeling anxiety compared with 27.3% (*n* = 12) who indicated continuing with anxiety. Therefore, after the intervention, there was an improvement in the average anxiety levels of the participants.

### Significant Differences Between Anxiety According to Sex

The data show statistically significant differences between girls (*Z* = −2.60, *p* < 0.009, η2 = 0.12) and boys (*Z* = −1.15, *p* < 0.251, η2 = 0.11) in the sample at the first sample collection (before relaxation techniques), with an intermediate effect size. The girls in the pre-test showed higher average mean values (23.8) than the boys (15.64).

## Discussion

After half a month of lockdown, the students began to show symptoms of anxiety due to the stress created by the lockdown itself (Qiu et al., 2020) and due to the stress of the end of the school year (Hernández-Pozo et al., 2008) (2019–2020). In addition to these factors, there is the stress of the new methodologies for teaching classes via telematics where ICTs prevail. Faced with this situation, the teaching staff decided to carry out an intervention with a group that had especially expressed high levels of anxiety.

In the present study, while the majority of the members of the groups have been women (84.1%), let us remember that the university population is currently composed mainly of women, and that the degree of Child Education is very feminized (Marquez-Domínguez et al., 2018).

Several studies have shown that alternatives to pharmacotherapy are effective in lowering anxiety levels (Aritzeta et al., 2017). Likewise, relaxation training, together with cognitive–behavioral techniques, is one of the most widely used procedures to reduce the symptoms of anxiety. The students in the present study received positively the possibility of practicing different relaxation techniques to lower their anxiety levels. Most of the students practiced Jacobson’s progressive relaxation (43.2%), followed by abdominal relaxation (36.4%), Schultz’s autogenic training (11.4%), and relaxation techniques through visualization (9%).

Jacobson’s technique was the most practiced because it is a simple technique to reach states of relaxation due to the awareness of bodily sensations of tension and distension. Likewise, Schultz’s autogenic training is one of the most effective relaxations for reducing anxiety levels (García et al., 2011), but it is also more complex and needs more practice by students.

In the present study, as in a recent study, students reported symptoms of anxiety in the face of the COVID-19 pandemic. Specifically, women in this study reported more anxiety than men. As with previous studies (Raya, 2009), the results of our study show the usefulness of relaxation techniques in reducing anxiety. However, although anxiety levels had decreased, there were still students in the post-test who were still anxious. For this reason, it was recommended that they continue to practice relaxation techniques after the study was completed.

In the discussion group, participants reported that practicing different relaxation techniques helped them decrease anxiety levels. Similarly, students said that learning these techniques in the current context of the crisis caused by the COVID-19 virus had been very appropriate for both academic learning and practical application. According to the results, at the beginning of the practice, they were not able to focus on these techniques, but as they practiced, the levels of relaxation improved considerably. This is due, as Amutio et al. (2015) point out, to the need for practice in the acquisition and improvement of relaxation techniques.

The learning of these techniques is beneficial not only for the present moment but also for your usual routine and your future profession. More than 20% of the students suffer from high levels of anxiety about university studies, and their performance is significantly reduced (Baeza et al., 2008). Similarly, it should be borne in mind that the situation of lockdown, fear, and uncertainty that the current pandemic seems to be creating may aggravate these anxiety levels (Qiu et al., 2020). Furthermore, we believe that it is especially significant that future teachers acquire the ability to manage anxiety since they will be referents and caregivers of children from 0 to 6 years old, a highly sensitive stage of development (López, 2005). Within this context, several studies show that teachers with lower levels of anxiety have a greater capacity to transmit well-being to students. In addition, calmer students develop a greater capacity for concentration and creativity and, in short, a greater ability to learn new content.

With this background, one of the main practical applications of this research has been to guide the effectiveness of non-pharmacological therapies, specifically relaxation techniques in a pandemic crisis situation, such as the one being experienced worldwide by COVID-19. This finding is very important taking into account that we are facing a population in which the consumption of anxiolytic drugs to combat anxiety is very high (Aznar et al., 2017), with women consuming the most. In this sense, it can be stated that psychoeducation about anxiety and training in relaxation techniques in a telematics way are new ways of dealing with anxiety symptoms and preventing the consumption of anxiolytics.

It should be noted that this research also has some limits that should be mentioned. Firstly, although the results obtained with the program carried out telematically have been satisfactory, the sample size is not significant. Secondly, the sample lacks homogeneous criteria since most of the participants are women. Thirdly, the study is mainly based on self-questionnaires. This may lead to confusion on the part of the participant when carrying out the study. Finally, there was no investigation into how anxiety symptoms were seen by other familial figure.

## Conclusion

The present research shows that relaxation techniques help to reduce the levels of anxiety that students may be experiencing when facing COVID-19.

On the other hand, telematics classes are an adequate way to reach this student body, and psychoeducation can be exercised through this means.

In short, it is important to take care of the mental health of the university students, and the effectiveness of a program created for a group of students at a university in northern Spain has been shown.

## Data Availability Statement

The raw data supporting the conclusions of this article will be made available by the authors, without undue reservation.

## Ethics Statement

The studies involving human participants were reviewed and approved by the Ethics Committee of the UPV/EHU. The participants provided their written informed consent to participate in this study.

## Author Contributions

AM, MG, and NO-E were involved in the conceptualization of the project and in the acquisition and analysis of the data. MD was involved in the interpretation of the data. All authors were involved in the drafting and revising of the work for intellectual content, provided approval for submission of the contents for publication, and agreed to be accountable for the accuracy and integrity of the project.

## Conflict of Interest

The authors declare that the research was conducted in the absence of any commercial or financial relationships that could be construed as a potential conflict of interest.
