# Corrigendum: Reduction of COVID-19 Anxiety Levels Through Relaxation Techniques: A Study Carried Out in Northern Spain on a Sample of Young University Students

**DOI:** 10.3389/fpsyg.2020.609098

**Published:** 2020-10-28

**Authors:** Naiara Ozamiz-Etxebarria, María Dosil Santamaría, Amaia Eiguren Munitis, Maitane Picaza Gorrotxategi

**Affiliations:** ^1^Department of Developmental and Educational Psychology, University of the Basque Country UPV/EHU, Leioa, Spain; ^2^Department of Research and Diagnostic Methods in Education, University of the Basque Country UPV/EHU, Leioa, Spain; ^3^Department of Didactics and School Organization, University of the Basque Country UPV/EHU, Leioa, Spain

**Keywords:** anxiety, relaxation techniques, university students, telematic psychoeducation, COVID-19

An author name was incorrectly spelled as ^**^Maria Dosil Santa María^**^. The correct spelling is ^**^Marí**a Dosil Santamar**ía^**^.

The authors apologize for this error and state that this does not change the scientific conclusions of the article in any way. The original article has been updated.

